# Screening for Hypoglycaemia Risk and Medication Changes in Diabetes Patients Using Pharmacy Dispensing Data

**DOI:** 10.3390/jcm13195855

**Published:** 2024-09-30

**Authors:** Indriastuti Cahyaningsih, Amal Asiri, Stijn de Vos, Jens H. J. Bos, Catharina C. M. Schuiling-Veninga, Katja Taxis, Petra Denig

**Affiliations:** 1 Department of PharmacoTherapy, -Epidemiology, and -Economics, University of Groningen, 9713 AV Groningen, The Netherlands; i.cahyaningsih@rug.nl (I.C.); a.asiri@rug.nl (A.A.); h.j.bos@rug.nl (J.H.J.B.); c.c.m.schuiling-veninga@rug.nl (C.C.M.S.-V.); k.taxis@rug.nl (K.T.); 2 Department of Pharmacist Professional Education, Faculty of Medicine and Health Sciences, Universitas Muhammadiyah Yogyakarta, Yogyakarta 55183, Indonesia; 3 Department of Pharmacy Practice, Faculty of Pharmacy, King Abdulaziz University, Jeddah 21589, Saudi Arabia; 4 Department of Clinical Pharmacy and Pharmacology, University of Groningen, University Medical Centre Groningen, 9713 AV Groningen, The Netherlands

**Keywords:** hypoglycaemia risk, primary care, glucose-lowering medications, treatment changes

## Abstract

**Background**: To improve hypoglycaemia management in primary care, more insight is needed into the opportunities to screen for hypoglycaemia risk and subsequent treatment modification using routinely available data. Our primary aim was to assess the number of diabetes patients with an estimated high risk of hypoglycaemia and describe the treatment changes in these patients using pharmacy dispensing data. Additionally, our aim was to investigate patient characteristics associated with such treatment changes. **Methods**: A drug utilisation cohort study with a 1-year follow-up using the IADB.nl pharmacy database was conducted. Patients aged 35 years or older who received at least two glucose-lowering medication dispensings in 2019 were included. Hypoglycaemia risk was determined using a validated algorithm based on patient demographics and dispensing data. The hypoglycaemia risk score ranged between 0 and 1. The anniversary method was used to evaluate treatment changes after 1 year. Factors associated with treatment changes were assessed by multinomial logistic regression. **Results**: Around one-quarter (26.9%) of the 36,628 included patients had a hypoglycaemia score of 0.6 or more. After a 1-year follow-up, the majority of these patients (88.9%) experienced no diabetes treatment changes. De-intensification was observed for 8.8% and intensification for 2.3%. Having a high-risk score, being female, and being younger in age were associated with de-intensification. **Conclusions**: A substantial number of primary care patients using glucose-lowering medications appear at risk of hypoglycaemia, whereas few of them undergo medication de-intensification. Pharmacy dispensing data can be helpful in screening for diabetes patients in whom a review of treatment is indicated.

## 1. Introduction

Hypoglycaemia is a complication often associated with intensive glucose control in patients with diabetes mellitus. Hypoglycaemia affects patients physically, psychosocially and financially, thus decreasing quality of life [1,2]. It is a well-recognised adverse event of treatment with insulin in patients with type 1 diabetes, but hypoglycaemia may receive less attention in patients with type 2 diabetes (T2D). Studies showed, however, that 10–30% of insulin-treated T2D patients [3,4,5] and 3–19% of T2D patients taking sulfonylureas may experience hypoglycaemia [6,7]. Many studies have been conducted in inpatient settings, but similar rates have been observed in primary care. For example, 13% of T2D patients treated with insulin and/or sulfonylurea in Dutch primary care settings experienced at least one hypoglycaemic event in the preceding year [8]. Also, it has been found that most hypoglycaemia events occur in outpatient settings [9].

In order to reduce the risk for hypoglycaemia events, it is important to monitor the medication treatment in relation to a range of clinical and patient-related factors. This is typically the responsibility of the physician managing the individual patient. In addition, pharmacists are involved in many countries to take the lead in conducting medication reviews intended to optimize treatment in selected populations [10]. Pharmacists could use screening tools making use of pharmacy dispensing data to select patients for such medication reviews. Many tools to assess hypoglycaemia risk have been developed to facilitate efficiently targeting populations to reduce hypoglycaemia events and improve patient safety [11,12,13,14,15]. These tools were mostly developed based on clinical outcomes or emergency department visits, data which are generally not available to pharmacists. To fill this gap, an algorithm has been developed to score the risk of hypoglycaemia in T2D patients using patient demographics and dispensing data available in community pharmacies [16]. This score can be used to screen for hypoglycaemia risk in primary care settings and identify patients who may require treatment de-intensification [17]. The process of de-intensification is also known as deprescribing [18]. A recent review indicated that pharmacists can play an important role in the process of deprescribing [19]. They can identify patients potentially in need of de-intensification and make clinicians, patients, and caregivers aware that reducing or ceasing medication is an option.

Currently, there is a need for additional actions to support treatment de-intensification among T2D patients, as the actual rates of treatment de-intensification after hypoglycaemia events appear to be relatively low [20]. One study found that rates of sulfonylurea and/or insulin de-intensification were less than 50% after hypoglycaemia-associated emergency visits or hospitalisations [21]. In a review, de-intensification rates among T2D patients with very low HbA1c levels (<42 mmol/mol) ranged from 20 to 45% [22]. In this review, it was noted that de-intensification rates could increase substantially when physicians receive reminders of patients reporting fear and/or symptoms of hypoglycaemia. Due to ageing or additional comorbidity, the benefits of strict hypoglycaemic control may decrease, whereas the risks for hypoglycaemic events increase [23]. However, there is no explicit guidance on which patients should receive treatment de-intensification. Several initiatives have been started to support treatment de-intensification in older and frail patients with T2D [24].

So far, not much is known about the number of patients that could be identified as having a high estimated risk of hypoglycaemia when applying the previously developed screening algorithm on pharmacy dispensing data. In addition, insight is lacking into the actual occurrence of de-intensification for glucose-lowering medication based on such data. Pharmacoepidemiologic studies can provide valuable information to assess drug use patterns [25]. In particular, drug utilisation studies are considered essential to facilitate rational drug use in society [26].

Therefore, we conducted a drug utilisation study aiming to (1) assess the number of patients with an estimated high risk of hypoglycaemia using a previously developed screening algorithm, (2) describe the treatment changes in glucose-lowering medications among these patients, and (3) investigate patient characteristics associated with treatment changes using a primary care pharmacy dispensing databases.

## 2. Materials and Methods

### 2.1. Study Design and Data Source

We conducted a drug utilisation cohort study with a 1-year follow-up. Data from the community pharmacy database IADB.nl at the University of Groningen were used (www.iadb.nl, accessed on 22 May 2023). This database includes outpatient medication dispensing data from more than 125 community pharmacies in the Netherlands and has been widely used and validated in the field of pharmacoepidemiology [27]. The database provides anonymous, individual records pertaining to filled dispensings, including drug Anatomical Therapeutic Chemical code(s) (ATC), prescription dates, and fill quantities. There is no information or data on medical conditions or on medications dispensed during hospital or inpatient stays. Studies using anonymised data are not subject to ethical approval in the Netherlands.

### 2.2. Study Population and Setting

To select the study population, the index date was defined as the date of the first dispensing of glucose-lowering medication(s) in 2019. The study population comprised all adults registered in the IADB for at least 5 years before and 1 year after the index date, aged 35 years or older at the index date, and receiving at least two glucose-lowering medication dispensings (ATC code: A10) between 1 January 2019 and 31 December 2019 (prevalent users). We excluded patients receiving only insulin prescriptions (A10A) in the preceding 5 years because those most likely have type 1 diabetes. This approach has been validated for pharmacoepidemiologic studies focusing on T2D patients [28].

In the Netherlands, it is common for T2D patients to visit their general practice for a regular checkup every three to four months. At such visits, HbA1c and/or glucose measurements, as well as other examinations, are conducted, and treatment and self-management are discussed. Depending on the findings, medication treatment can be adjusted.

### 2.3. Outcome Variables

#### 2.3.1. Hypoglycaemia Risk Score

To screen for patients at high risk of hypoglycaemia, we applied an algorithm which has been developed previously by Crutzen et al. (2021) [16]. This algorithm was validated in a primary care population, which was similar to the population in the present study. The validation process made use of extensive clinical data, including data on actual hypoglycaemia events as documented in the medical record by a diagnostic code or a glucose measurement below 3.9 mmol/L. The algorithm based on dispensing data was compared to an algorithm including clinical data, such as eGFR, HbA1c, total cholesterol, high blood pressure, albuminuria, body weight, depression, hypercholesterolaemia, and non-chronic infection. The addition of clinical data, however, did not improve the algorithm’s performance, as both models had similar and acceptable AUC values. Therefore, the algorithm with demographic and medication use data only was suitable for our study to estimate the risk of hypoglycaemia using dispensing data [16].

The algorithm includes ten variables: the patient’s sex, age, total medication count, glucose-lowering medication count (A10), sulfonylurea use (A10BB), insulin use (A10A), premixed insulin use (A10AD), insulin count (A10A), antidepressant use (N06A), and insulin use duration capped at 5 years (A10A). Both medication use and medication count were assessed using the medication dispensing date at or around the index date with a time window of plus or minus 45 days to capture all dispensings within a 90-day period, which is the common duration for chronic medications.

The hypoglycaemia risk scores were calculated to fall within a range between 0 and 1. Patients were designated to one of two categories: low or high risk. A score of 0.6 was determined to be the cut-off point between the two categories, with the assumption that patients with hypoglycaemia risk scores of 0.6 or higher were more likely to be in need of glucose-lowering medication de-intensification. This cut-off point was based on a previous study conducted by Crutzen et al. (2023) [17].

#### 2.3.2. Medication Changes

We classified glucose-lowering medications into two groups according to their potential to cause hypoglycaemia, namely: “hypoglycaemia” and “other” medications. Hypoglycaemia medications include medications that can cause severe hypoglycaemia as stated in the Dutch guideline for the management of T2D, that is, sulfonylureas and insulins. Other medications include all other glucose-lowering medications [29] (for the full list, see Table S1).

The main outcome was the change in dispensed hypoglycaemia medication at 1-year follow-up compared to the index date. The following definitions were used: (1) no change: no intensification or de-intensification of glucose-lowering medication; (2) de-intensification: switching from insulin to non-insulin glucose-lowering medication, switching from sulfonylureas to other non-insulin glucose-lowering medications, or discontinuation of hypoglycaemia medications; (3) intensification: switching from non-insulin glucose-lowering medication to insulin, switching from other non-insulin glucose-lowering medications to sulfonylurea, or the addition of hypoglycaemia medications (Table S1). Dosing was not taken into account. Although dose de-intensification of insulins is a relevant action to reduce hypoglycaemic events, this cannot be captured with dispensing data. It is common for the dose of insulin to be adapted as needed, and information on actual use is not systematically documented in the database.

**Figure 1:**
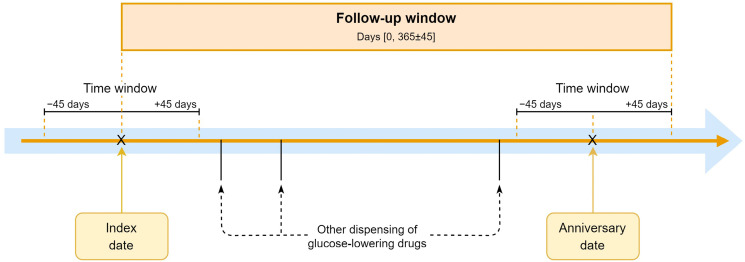
The anniversary method.

We used the anniversary method to assess treatment changes at 1-year follow-up, allowing for sufficient time to implement medication changes. The anniversary date is defined as 365 days from the index date. We again applied a time window of plus or minus 45 days to capture prescriptions at or around this date (Figure 1).

### 2.4. Statistical Analysis

Baseline characteristics of the study participants, hypoglycaemia risk scores and treatment changes were reported using means with standard deviations (SD) or medians with interquartile ranges (IQR), depending on whether continuous variables were normal or non-normally distributed. For categorical variables, the number and percentages of patients were reported.

We used multinomial logistic regression to estimate the association between hypoglycaemia risk and treatment changes corrected for age and sex; treatment changes (no change as reference, de-intensification, and intensification), hypoglycaemia risk category (low-risk as reference, high-risk), sex (male as reference, female) and age (as continuous data). Sensitivity analyses were performed with four different time windows at index and anniversary date (±14 days, ±30 days, ±45 days, and ± 60 days) to determine the impact of time windows on treatment changes. Analysis was conducted using R (version 4.0.3; The R Foundation for Statistical Computing Platform, Vienna, Austria) with the R package nnet. The statistical significance threshold was a two-sided *p* = 0.05.

## 3. Results

### 3.1. Patient Characteristics at the Index Date

**Figure 2:**
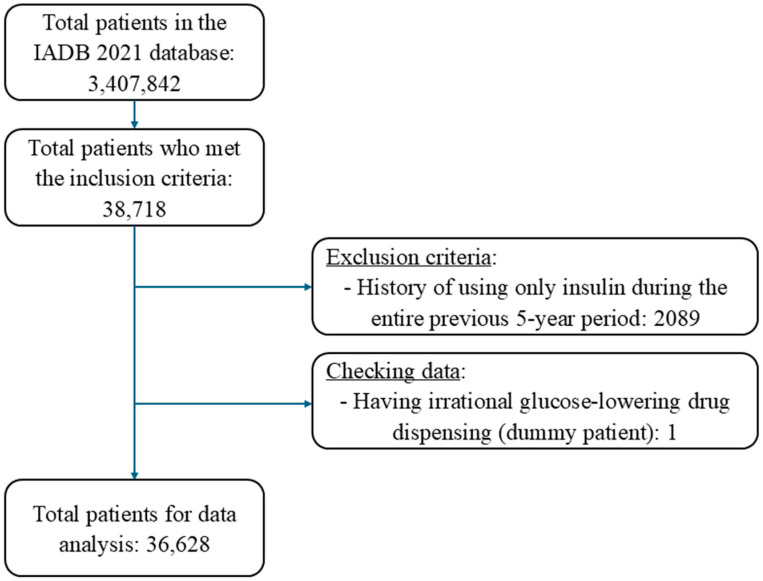
Patient inclusion flow chart.

Overall, 36,628 patients fulfilled the inclusion criteria out of 3,407,842 patients recorded in the IADB.nl database (Figure 2). The average age of the included patients was 67.8 years (SD: 11.4), and 53% were male, with little difference between high-risk and low-risk patients (Table 1). The median number of medications dispensed per patient at the index date was 7 (IQR: 5–10). As could be expected, this total number was higher in the high-risk (9, IQR: 7–13) compared to the low-risk patients (7, IQR: 5–10). Almost all high-risk patients received insulin, either as monotherapy or combination therapy with non-insulin medications, and the mean duration of insulin use was 3.3 years (Table 1). Only very few low-risk patients received insulin treatment and instead commonly received metformin as a monotherapy (55.1%). Sulfonylurea medications were used either as monotherapy or in combination by 22.5% of patients in the high-risk categories and 41.8% of patients in the low-risk categories (Table 1).

### 3.2. Hypoglycaemia Risk Score and Medication Changes

**Figure 3:**
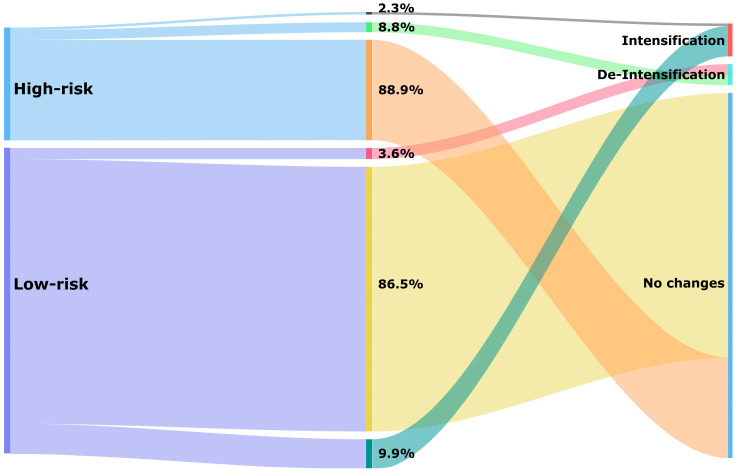
Treatment changes of glucose-lowering medications based on hypoglycaemia risk category after a 1-year follow-up period.

Just over a quarter of patients had a high hypoglycaemia risk score (26.9%). The median score was 0.85 (IQR: 0.79–0.88) in the high-risk and 0.39 (IQR: 0.35–0.48) in the low-risk categories. Most patients experienced no change after a 1-year follow-up period (87.0%), and within the high-risk category, almost ninety per cent of patients (88.9%) had no change in the hypoglycaemia medications used at the 1-year follow-up (Figure 3). Particularly noteworthy was the substantial group with an estimated high risk who were on a combination of insulin and sulfonylurea (17.1%) that experienced no change (Table 2).

De-intensification of hypoglycaemia medication occurred in 5.0% of all patients (Table 2). In the high-risk category, 8.8% received de-intensification of treatment compared to 3.6% in the low-risk category (Figure 3). Treatment de-intensification in the high-risk category of patients included the discontinuation of insulin (5.7%), discontinuation of sulfonylurea (2.3%), switching from insulin to non-insulin (0.5%), and switching from sulfonylurea to other non-insulin medications (0.3%) (Table 2).

Intensification of hypoglycaemia medication occurred in 8.3% of all patients. This percentage was higher in the low-risk category (9.9%) compared to the high-risk category (2.3%) (Figure 3). Among high-risk patients, the addition of sulfonylurea was the most common change observed (1.2%). Additionally, switching from non-insulin to insulin (1.0%) or switching from other non-insulin medications to sulfonylurea (0.1%) was seen among these patients (Table 2).

The sensitivity analyses showed that using shorter time windows resulted in higher rates of de-intensification, particularly due to an increase in patients being classified as discontinuing insulin (Table S2). This illustrates that insulin is particularly prone to misclassification since, with a widening of the time interval, it was observed that the insulin dispensings had continued. The largest misclassification was seen when using a time window of 14 days, whereas only a few very small differences were noted between 45 and 60 days.

De-intensification of hypoglycaemia medication was significantly associated with having a high hypoglycaemia risk score (OR: 2.34; 95% CI: 2.13–2.57), female sex (OR: 1.15; 95% CI: 1.03–1.25), and lower age (OR: 0.98; 95% CI: 0.98–0.99). On the other hand, the intensification of such medication was associated with a younger age and low-hypoglycaemia risk score (Table 3).

## 4. Discussion

Based on a dispensing-based screening algorithm, around one-quarter of the included patients with T2D medication treatment had a high estimated hypoglycaemia risk, making them eligible for treatment review. After the 1-year follow-up, less than 9% of these estimated high-risk patients had experienced de-intensification of hypoglycaemia medications, that is, discontinuation of insulin and/or sulfonylureas. The majority of these high-risk patients experienced no change regarding such medication. Having a high-risk score, being female, and being younger in age were all associated with receiving de-intensification.

The number of patients estimated at risk of hypoglycaemia in this study is substantial. One previous study which used the same algorithm to estimate hypoglycaemia risk showed that 60% of T2D patients with a high hypoglycaemia risk score actually experienced hypoglycaemia at some time in the past, with 15% in the last 4 weeks [17]. We observed that patients with a high hypoglycaemia risk score were more likely to have their hypoglycaemia medication de-intensified, but with 9%, this de-intensification rate was low. Other studies observed rates of at least 20% among adult T2D patients with low HbA1c levels, who may have been eligible for de-intensification [22]. These studies, however, included discontinuation of any glucose-lowering medication in their assessments, whereas we considered discontinuation of hypoglycaemia medications only. In studies focusing on adult T2D patients, the high risk of hypoglycaemia needs to be balanced against the beneficial effects of intense glycaemic control. In cases of high hypoglycaemia risk, de-intensification of sulfonylurea or insulin while intensifying other glucose-lowering medication(s) can thus be necessary and appropriate. This factor was taken into account in our treatment modification definitions. The new generation of glucose-lowering medications, such as glucagon-like peptide 1 receptor agonists (GLP-1RA), dipeptidyl peptidase-4 (DPP-4) inhibitors and sodium-glucose cotransporter 2 (SGLT-2) inhibitors may pose a lower risk for hypoglycaemia [30]. We observed dispensing of these medications in very few patients during our study period. The majority of T2D patients managed in primary care were still treated with older-generation medications. This is likely due to the fact that until the end of 2021, the Dutch guideline for management of T2D recommended the newer medication as second-line treatment. Since 2022, SGLT-2 inhibitors and GLP-1RA have been recommended as first-line treatment only for T2D patients with very high cardiovascular and/or renal risks.

The weighing of benefits and risks should depend on certain individual patient characteristics, such as comorbidity, frailty, or life expectancy [31]. Previously, it was observed that de-intensification rates were somewhat higher in patients who demonstrated more comorbidity features or who had a lower life expectancy, but age and sex had no consistent impact on these rates [22]. De-intensification rates were found to be higher among very old and frail people in long-term care facilities [32]. Our study adds to these findings as that both de-intensification and intensification were more likely among younger patients at high risk of hypoglycaemia. This suggests that any kind of treatment adjustment is more likely in younger patients, and this is not necessarily driven by hypoglycaemia risk. Finally, in our study, female patients were more likely to undergo de-intensification. This is relevant since it has been observed that insulin-treated females, in particular, were more likely to experience hypoglycaemia [33,34]. This has been attributed to lower metabolic responses to hypoglycaemia and higher insulin dosages due to body weight considerations in females compared to males [33,34]. Previous studies involving other drugs indicate that women more often report adverse drug reactions than men, which could be linked to receiving too high dosages and physiological differences but also to gender-related differences in experiencing and reporting events [35].

### 4.1. Strengths and Limitations

This study has several strengths. Firstly, the algorithm was developed for the same setting and population to assess hypoglycaemia risk, that is, T2D patients in primary care. This algorithm is intended to identify any patients at high risk at an index date, thereby useful as a screening tool for initiatives focusing on proactive medication de-intensification in primary care. Secondly, we were able to assess medication changes after a 1-year period to allow sufficient time to implement de-intensification. Within a 1-year period, most patients have visited the general practice at least three times for their regular checkups. It should be noted, though, that high-risk patients, as assessed with the hypoglycaemia risk score, may have already started hypoglycaemic medication sometime before the index date.

In terms of limitations, the most important one is that dose reductions could not be reliably captured by using pharmacy dispensing data. A previous study showed that the percentage of patients with dose reductions for sulfonylureas after hypoglycaemia was small (5%) [21]. In that study, dose changes for insulin could also not be captured. It is to be expected that part of the population classified as having received no treatment modification did experience changes in insulin dosing. Secondly, we classified medication according to hypoglycaemia risk as determined by the Dutch guidelines and considered switching from insulin to any non-insulin medication as de-intensification. These non-insulin medications do include medication that can cause hypoglycaemia. Thirdly, we used the anniversary method with a time window of 90 days to determine medications prescribed at the index and anniversary date. This may result in some misclassifications due to medication switches occurring within the time window. There were a few patients (less than 1%) who received more than one sulfonylurea or more than two insulins within the 90-day window at the index date, indicative of a medication switch within this time window.

Furthermore, the association between patient characteristics and treatment changes should not be interpreted as causal, also considering that other factors not included in this study may influence treatment changes, most notably a patient’s blood glucose level and any diabetes-related comorbidities. This is the consequence of dispensing data from pharmacies that do not include such information. For comparison, though, de-intensification rates were also presented for those with low-risk scores, indicative of baseline rates of treatment changes. A final weakness could be that this study included patients over 35 years old and excluded patients who had received only insulin for the past 5 years in order to identify T2D patients. By using this selection method, we may have excluded a small number of younger T2D patients and included some patients with other types of diabetes. It is estimated that the proportion of T2D patients who are younger than 35 years is only less than 1 per 1000 [36].

### 4.2. Implications for Practice and Research

Almost all of the high-risk patients in our study population were on insulin treatment, whereas sulfonylurea treatment was more common in patients from the low-risk category. In the previous study that used the same algorithm to identify patients that might be eligible for de-intensification, 90% of the selected high-risk patients used insulin and over 60% used sulfonylureas. In that study, however, a preselection was made for patients 45 years and older who were using insulins and/or sulfonylureas [17]. Our study demonstrated that in patients 35 years and older who were using any glucose-lowering medications, most patients on insulin would end up in the high-risk group. This suggests that the algorithm is particularly valuable in identifying those patients taking sulfonylureas who are at high risk of hypoglycaemia.

Decisions to de-intensify or intensify glucose-lowering medications are likely to be driven by HbA1c or glucose levels on the one hand and experiencing hypoglycaemic symptoms on the other [22,23]. Therefore, not all patients with a high hypoglycaemia risk score are actually in need of treatment de-intensification. Screening for patients with a high hypoglycaemia risk score using information routinely available in primary healthcare settings, however, is relevant for clinical practice. The dispensing data available in community pharmacies can thus be used to support the initiation of the deprescribing process, which is seen as a meaningful role for pharmacists [19]. A previous pharmacist-led intervention showed that sulfonylurea and/or insulin could be de-intensified in 18% of the patients with high hypoglycaemia risk, which is more than double the percentage observed in our study [17]. This suggests that healthcare providers in primary care need to put more effort into regularly assessing the hypoglycaemia burden and adjusting medication treatment to minimize the risk of hypoglycaemia, as suggested by diabetes guidelines [37,38]. Additionally, a previous review illustrated that both healthcare providers and patients often experience barriers to de-intensify or deprescribe glucose-lowering medication, and this is something that should be addressed to support this process [39].

Future research could focus on hypoglycaemia medications prescribed for high-risk patients in other practice settings and on including longer follow-up data to assess how risks may change over time. Additionally, studies are needed to assess whether hypoglycaemia events can be reduced by applying a screening tool for hypoglycaemia risk in routine care. Finally, studies including more comprehensive clinical information are also needed to estimate what would be the optimal de-intensification rate among patients with an estimated high risk of hypoglycaemia.

## 5. Conclusions

A substantial number of primary care patients using glucose-lowering medications may be at risk of hypoglycaemia, whereas few appear to undergo treatment de-intensification. Assessing the risk of hypoglycaemia with data routinely available in pharmacies can help healthcare providers identify patients at increased risk and consider the need for treatment de-intensification.

## Figures and Tables

**Table 1:** Characteristics and treatment of patients at index date.

Characteristics	High-Risk Category		Low-Risk Category		Total	
	*n*	%	*n*	%	*n*	%
Number of patients	9867	26.9	26,761	73.1	36,628	100
Risk score (median [IQR])	0.85 (0.79–0.88)		0.39 (0.35–0.48)		0.46 (0.36–0.74)	
Age (mean (SD)), years ^ǂ^	67.9 (11.3)		67.8 (11.4)		67.8 (11.4)	
Sex ^ǂ^						
Female	4757	48.2	12,462	46.6	17,219	47.0
Male	5110	51.8	14,299	53.4	19,409	53.0
Total medication count (median [IQR]) ^ǂ^	9 [7–13]		7 [5–10]		7 [5–10]	
Glucose-lowering medication count (median [IQR]) ^ǂ^	3 [2–3]		1 [1–2]		2 [1–2]	
Insulin count^ǂ^						
0	2	0.0	26,755	100	26,757	73.1
1	5573	56.5	6	0.0	5579	15.2
2 or more	4292	43.5	-	-	4292	11.7
Insulin duration (mean (SD), years ^ǂ^	3.3 (1.6)		-	-	0.9 (1.7)	
Sulfonylurea count ^ǂ^						
0	7068	71.6	15,574	58.2	22,642	61.8
1 or more	2799	28.4	11,187	41.8	13,986	38.2
Premixed insulin use ^ǂ^						
Yes	1894	19.2	-	-	1894	5.2
No	7973	80.8	26,761	100	34,734	94.8
Antidepressant use ^ǂ^						
Yes	1493	15.1	3237	12.1	4730	12.9
No	8374	84.9	23,524	87.9	31,898	87.1
Therapy type for diabetes						
Monotherapy	660	6.7	16,575	62	17,235	47.1
Combination therapy	9207	93.3	10,186	38	19,393	52.9
Glucose-lowering medication classes						
Monotherapy						
Insulin	660	6.7	5	0.0	665	1.8
Metformin	-	-	14,750	55.1	14,750	40.3
Sulfonylureas	-	-	1582	5.9	1582	4.3
Alpha glucosidase inhibitors	-	-	34	0.1	34	0.1
Thiazolidinediones	-	-	7	0.0	7	0
DPP4 inhibitors	-	-	79	0.3	79	0.2
GLP-1 agonist	-	-	36	0.1	36	0.1
SGLT2 inhibitors	-	-	11	0.0	11	0.0
Other glucose-lowering medications	-	-	2	0.0	2	0.0
Combination therapy						
Insulin combination	4292	43.5	-	-	4292	11.7
Insulin + sulfonylureas	2223	22.5	0	0.0	2223	6.1
Insulin + other medication	2690	27.3	1	0.0	2691	7.3
Non-insulin combination, including SU	2	0.0	9606	35.9	9608	26.2
Non-insulin combination without SU	0	0.0	648	2.4	648	1.8

Notes: SD—Standard Deviation; IQR—Interquartile range; DPP4—Dipeptidyl peptidase-4; GLP-1—Glucagon-like peptide 1; SGLT2—Sodium-glucose cotransporter-2; SU—Sulfonylurea; ^ǂ^—variables of the hypoglycaemia algorithm.

**Table 2:** Treatment changes of glucose-lowering medications according to hypoglycaemia risk score categories.

Treatment Changes	High-Risk Category (*n* = 9867)		Low-Risk Category (*n* = 26,761)		Total (*n* = 36,628)	
	*n*	%	*n*	%	*n*	%
* No changes (monotherapy)*						
No modification of insulin	557	5.6	5	0.0	562	1.5
No modification of sulfonylurea	-	-	1185	4.4	1185	3.2
No modification of other medication	-	-	12,400	46.3	12,400	33.9
Discontinuation of other medication	-	-	743	2.8	743	2.0
Addition of other medication to insulin	54	0.5		-	54	0.1
Addition of other medication to sulfonylurea	-	-	180	0.7	180	0.5
Addition of other medication to other medication	-	-	123	0.5	123	0.3
* No changes (combination therapy)*						
No modification of insulin combination	3890	39.4	-	-	3890	10.6
No modification of insulin + sulfonylurea	1683	17.1	-	-	1683	4.6
No modification of insulin + other medication	2303	23.3	-	-	2303	6.3
No modification of other medication combination	-	-	369	1.4	369	1.0
No modification of sulfonylurea + other medication	1	0.0	7749	29.0	7750	21.2
Discontinuation of other medications	288	2.9	369	1.4	657	1.8
Addition of other medication	-	-	12	0.0	12	0.0
**Subtotal**	**8776**	**88.9**	**23,135**	**86.5**	**31,911**	**87.0**
* Intensification (monotherapy)*						
Addition of insulin	-	-	303	1.1	303	0.8
Addition of sulfonylurea	9	0.1	1359	5.1	1368	3.7
Switching non-insulin to insulin	-	-	27	0.1	27	0.1
Switching other medication to sulfonylurea	-	-	143	0.5	143	0.4
* Intensification (combination therapy)*						
Addition of insulin	-	-	542	2.0	542	1.5
Addition of sulfonylurea	108	1.1	115	0.4	223	0.7
Switching non-insulin to insulin	100	1.0	143	0.5	243	1.0
Switching other medication to sulfonylurea	5	0.1	19	0.1	24	0.1
**Subtotal**	**222**	**2.3**	**2651**	**9.9**	**2873**	**8.3**
* De-intensification (monotherapy)*						
Switching insulin to non-insulin	15	0.1	-	-	15	0.0
Switching sulfonylurea to another medication	-	-	29	0.1	29	0.1
Discontinuation of sulfonylurea	-	-	78	0.3	78	0.2
Discontinuation of insulin	25	0.3	-	-	25	0.1
* De-intensification (combination therapy)*						
Switching insulin to non-insulin	37	0.4	-	-	37	0.1
Switching sulfonylurea to another medication	26	0.3	60	0.2	86	0.2
Discontinuation of sulfonylurea	230	2.3	808	3.0	1038	2.8
Discontinuation of insulin	536	5.4	-	-	536	1.5
**Subtotal**	**869**	**8.8**	**975**	**3.6**	**1844**	**5.0**

**Table 3:** Factors associated with treatment de-intensification and intensification.

Predictors		Odds Ratios (95%CI)	
		De-Intensification ^ǂ^	Intensification ^ǂ^
Hypoglycaemia risk category	Low-risk	Reference	Reference
	High-risk	2.34 (2.13–2.57)	0.22 (0.19–0.25)
Sex	Male	Reference	Reference
	Female	1.15 (1.03–1.25)	1.01 (0.94–1.11)
Age	Years	0.98 (0.98–0.99)	0.98 (0.97–0.98)

Notes: ^ǂ^—Assessed by multinomial logistic regression with no change as a reference outcome group.

## Data Availability

Data are contained within the article and Supplementary Materials. The original dataset is not publicly available due to the IADB data protection policy. Request to access the data should be directed to the corresponding author upon reasonable request.

## Supplementary Materials

The following supporting information can be downloaded at: https://www.mdpi.com/article/10.3390/jcm13195855/s1, Table S1 Operational definitions of treatment changes; Table S2 Comparison of treatment changes using different time windows.
