# Supplementary material for: Screening for Hypoglycaemia Risk and Medication Changes in Diabetes Patients Using Pharmacy Dispensing Data
Source: J Clin Med. 2024 Sep 30;13(19):5855. doi: 10.3390/jcm13195855 (PMC11477424; doi:10.3390/jcm13195855)
Supplement: Supplementary file 1 [file jcm-13-05855-s001.zip › jcm-3186457-supplementary.pdf]

Table S1. Operational definitions of treatment changes of glucose-lowering drug based on the risk of medication to cause hypoglycaemia

Treatment changes	Monotherapy	Combination therapy
No Change	1. No modification of glucose-lowering drug (GLD) classes.<br>- Insulin → insulin<br>- Sulfonylurea (SU) → SU<br>- Other medication → other medication.<br>2. Discontinuation of any other medication.<br>3. Addition of any other medications.	1. No modification of GLD(s) classes.<br>- Insulin combination → insulin combination<br>- Insulin + SU → insulin + SU<br>- Insulin + other medication→ insulin + other medication<br>- SU combination → SU combination<br>- Other medication combination → Other medication combination<br>- SU + other medication → SU + other medication<br>2. Discontinuation of any other medication.<br>3. Addition of any other medications.
De-intensification	1. Switching insulin to non-insulin GLD.<br>2. Switching SU to other medication.<br>3. Discontinuation of SU or insulin.	1. Switching insulin to non-insulin GLD.<br>- Insulin + non-insulin GLD → non-insulin GLD combination<br>- Insulin combination + non-insulin GLD → insulin + non-insulin GLD combination<br>2. Switching SU to other medication.<br>- SU + other medication→ other medication combination<br>- SU + insulin → other medication + insulin<br>3. Discontinuation of SU or insulin.
Intensification	1. Switching non-insulin GLD to insulin.<br>2. Switching other medication to SU.<br>3. Addition of SU or insulin.	1. Switching non-insulin GLD to insulin.<br>- Non-insulin GLD combination → Insulin + non-insulin GLD<br>2. Switching other medication to SU.<br>- Other medication combination → SU + other medication<br>- Insulin + other medication → insulin + SU<br>3. Addition of SU or insulin.

Notes

Abbreviations	:	GLD, glucose-lowering drugs, SU, Sulfonylurea
Index date	:	The date of the first prescription of GLD (A10) in 2019 with the time window (± 45 days from the index date).
Follow-up period	:	The GLD (A10) prescribed within 365 days after the index date (anniversary date) with the time window (± 45 days from the anniversary date).
Hypoglycaemia medications	:	Sulfonylureas, insulins (according to NHG guideline 2021).
Other medications	:	Metformin, Alpha glucosidase inhibitors, Thiazolidinediones, Dipeptidyl peptidase 4 (DPP-4) inhibitors, Glucagon-like peptide-1 (GLP-1) analogues, Sodium-glucose co-transporter 2 (SGLT2) inhibitors, other blood glucose-lowering drugs or fixed-dose combinations of any of these drugs, excl. insulins and sulfonylureas.

Table S2. Comparison of treatment changes of glucose-lowering medications using the time window of ± 14 days, ± 30 days, ± 45 days and ± 60 days (Sensitivity analysis)

	**High-risk category**				**Low-risk category**			
	**± 14 days (n=9346)**	**± 30 days (n=9682)**	**± 45 days (n=9867)**	**± 60 days (n=10,000)**	**± 14 days (n=27,282)**	**± 30 days (n=26,946)**	**± 45 days (n=26,761)**	**± 60 days (n=26,628)**
	**n (%)**	**n (%)**	**n (%)**	**n (%)**	**n (%)**	**n (%)**	**n (%)**	**n (%)**
**No changes (monotherapy)**								
No modification of insulin	614 (6.6)	586 (6.1)	557 (5.6)	531 (5.3)	7 (0.0)	6 (0.0)	5 (0.0)	4 (0.0)
No modification of SU	-	-	-	-	1222 (4.5)	1201 (4.5)	1185 (4.4)	1161 (4.4)
No modification of other medication	-	-	-	-	12,482 (45.8)	12,442 (46.2)	12,400 (46.3)	12,352 (46.4)
Discontinuation of other medication	-	-	-	-	1083 (4.0)	856 (3.2)	743 (2.8)	665 (2.5)
Addition of other medication to insulin	76 (0.8)	55 (0.6)	54 (0.5)	49 (0.5)	-	-	-	-
Addition of other medication to SU	-	-	-	-	245 (0.9)	201 (0.7)	180 (0.7)	173 (0.6)
Addition of other medication to other medication	-	-	-	-	143 (0.5)	121 (0.4)	123 (0.5)	119 (0.4)
**No changes (combination therapy)**								
No modification of insulin combination	3777 (40.4)	4132 (42.6)	4373 (44.3)	4527 (45.3)	-	-	-	-
No modification of insulin + SU	1284 (13.7)	1427 (14.7)	1504 (15.2)	1560 (15.6)	-	-	-	-
No modification of insulin + other medication	1851 (19.8)	1965 (20.3)	1999 (20.3)	2001 (20.0)	-	-	-	-
No modification of other medications	-	-	-	-	357 (1.3)	366 (1.4)	369 (1.4)	374 (1.4)
No modification of SU + other medication	-	1 (0.0)	1 (0.0)	1 (0.0)	7341 (26.9)	7598 (28.2)	7749 (29.0)	7836 (29.4)
Discontinuation of other medication	297 (3.2)	295 (3.0)	288 (2.9)	288 (2.9)	424 (1.6)	376 (1.4)	369 (1.4)	387 (1.5)
Addition of other medication	-	-	-	-	5 (0.0)	10 (0.0)	12 (0.0)	13 (0.0)
**Sub total**	**7899 (84.5)**	**8460 (87.3)**	**8776 (88.8)**	**8957 (89.6)**	**23,309 (85.5)**	**23,177 (86.0)**	**23,135 (86.5)**	**23,084 (86.6)**
**Intensification (monotherapy)**								
Addition of insulin	-	-	-	-	52 (0.2)	358 (1.3)	303 (1.1)	550 (2.1)
Addition of SU	29 (0.3)	18 (0.2)	9 (0.1)	9 (0.1)	1383 (5.1)	1387 (5.1)	1359 (5.1)	1338 (5.0)
Switching non-insulin to insulin	-	-	-	-	404 (1.5)	31 (0.1)	27 (0.1)	21 (0.1)
Switching other medication to SU	-	-	-	-	198 (0.7)	165 (0.6)	143 (0.5)	120 (0.5)
**Intensification (combination therapy)**								

Addition of Insulin	-	-	-	-	418 (1.5)	523 (1.9)	542 (2.0)	550 (2.0)
Addition of SU	97 (1.0)	97 (1.0)	108 (1.1)	107 (1.1)	100 (0.4)	106 (0.4)	115 (0.4)	124 (0.5)
Switching non-insulin to insulin	-	100 (1.0)	100 (1.0)	96 (1.0)	263 (0.9)	166 (0.6)	143 (0.5)	134 (0.5)
Switching other medication to SU	11 (0.1)	8 (0.1)	5 (0.1)	4 (0.0)	21 (0.1)	18 (0.1)	19 (0.1)	20 (0.1)
**Sub total**	**137 (1.4)**	**223 (2.3)**	**222 (2.3)**	**216 (2.2)**	**2839 (10.4)**	**2754 (10.1)**	**2651 (9.8)**	**2585 (9.8)**
**Deintensification (monotherapy)**								
Switching insulin to non-insulin	33 (0.3)	23 (0.3)	15 (0.1)	11 (0.1)	-	-	-	-
Switching SU to other medication	-	-	-	-	44 (0.2)	33 (0.1)	19 (0.1)	26 (0.1)
Discontinuation of SU	-	-	-	-	145 (0.5)	102 (0.4)	78 (0.3)	75 (0.3)
Discontinuation of insulin	92 (1.0)	41 (0.4)	25 (0.3)	16 (0.2)	-	-	-	-
**Deintensification (combination therapy)**								
Switching insulin to non-insulin	58 (0.6)	48 (0.5)	37 (0.4)	30 (0.3)	-	-	-	-
Switching SU to other medication	25 (0.3)	24 (0.2)	26 (0.3)	28 (0.3)	64 (0.2)	60 (0.2)	60 (0.2)	64 (0.2)
Discontinuation of SU	190 (2.0)	208 (2.1)	230 (2.3)	248 (2.5)	881 (3.2)	819 (3.2)	808 (3.0)	794 (3.0)
Discontinuation of insulin	912 (9.8)	655 (6.8)	536 (5.4)	494 (4.9)	-	1 (0.0)	-	-
**Sub total**	**1310 (14.0)**	**999 (10.3)**	**869 (8.8)**	**827 (8.3)**	**1134 (4.1)**	**1015 (3.9)**	**965 (3.6)**	**959 (3.6)**

Abbreviations : SU, Sulfonylurea
